# Diagnosis and management of face presentation: a case report featuring an innovative diagnostic approach and fetal spinal protection technique

**DOI:** 10.3389/fmed.2025.1664796

**Published:** 2025-09-26

**Authors:** Ying Cui, Jingjing Yi, Birong Xiao, Chen Chen

**Affiliations:** Department of Obstetrics and Gynecology, Affiliated Hospital of Chengdu University of Traditional Chinese Medicine, Deyang People’s Hospital, Deyang, China

**Keywords:** case report, face presentation, diagnosis, vaginal dilator, bimanual guidance method

## Abstract

We report the case of a 40 + 2-week pregnant woman who experienced spontaneous rupture of membranes at the end of the first stage of labor, followed by palpation of an irregular, soft tissue mass at the presenting part. Initial examination suggested a breech presentation; however, a definitive diagnosis could not be established by palpation alone. Using a vaginal dilator to directly visualize the presenting part, we confirmed the diagnosis of face presentation and achieved a successful vaginal delivery, with favorable outcomes for both the mother and the neonate. By reviewing the patient’s medical history, diagnostic process, and delivery progression, we identified the causative factors and underlying delivery mechanism in this case. We propose a simple and practical diagnostic approach and share a novel obstetric technique for fetal spinal protection during delivery. Furthermore, we underscore the importance of multidisciplinary collaboration in the management of facial presentation to optimize maternal and neonatal outcomes, thereby offering a reference for the clinical management of similar cases.

## 1 Introduction

Malposition of the fetus is a major cause of dystocia and a frequent indication for obstetric intervention during labor. Among these abnormalities, persistent occiput posterior position is the most common, occurring in approximately 5.2% of deliveries. Breech presentation is observed in approximately 3.1% of cases, while transverse lie is less frequent, with an incidence of approximately 0.12% (1). Face presentation is the rarest form, occurring in only 0.014% of deliveries (2). Face presentation refers to a fetal position in which the face, from the forehead to the chin, constitutes the presenting part during descent through the birth canal. This condition is characterized by marked hyperextension of the fetal head, such that the occiput may lie near the fetal back. Face presentation is classified into mentum anterior, mentum posterior, and mentum transverse. Among these, approximately three-quarters of mentum anterior cases may be considered for vaginal delivery, whereas mentum posterior presentation most often requires cesarean delivery (3, 4).

The primary method for the clinical diagnosis of a face presentation is a vaginal digital examination. A definitive diagnosis is achieved by palpating facial cranial landmarks—such as the orbital ridges, orbits, nasal bridge, oral fissure, and chin—during the first or second stage of labor. Ultrasonography serves as a valuable adjunct or alternative modality for confirmation (5, 6). Sonographic findings typically include hyperextension of the fetal cervical spine, an S-shaped spinal curvature, and an occipitocervical angle of less than 90° (1, 7, 8). However, pelvic bone shadows and soft tissue artifacts limit the quantitative accuracy of ultrasonography. Furthermore, the scarcity of cases restricts clinicians’ palpation experience, while continuous fetal movement further complicates diagnosis. Consequently, the rate of missed and misdiagnosed cases remains high, with approximately 44.4% confirmed only during cesarean delivery, underscoring the considerable diagnostic challenge of this condition (2).

Established risk factors for face presentation include fetal anomalies (e.g., anencephaly), multiparity, and polyhydramnios. Additionally, any condition that impedes fetal head flexion or promotes neck extension—such as multiple nuchal cord loops, cephalopelvic disproportion, or pelvic contracture—can increase the risk of face presentation (9). Improper management of face presentation can result in serious maternal and fetal complications, including uterine rupture, severe perineal lacerations, fetal spinal injury, and neonatal mortality (2, 10), posing significant challenges for obstetricians. Data indicate that 89% of clinicians prefer cesarean delivery in such cases, a rate approximately three times higher than that for vertex presentations (2, 11). This preference is partly due to the complexity of face presentations—often accompanied by prolonged labor and altered fetal heart rate patterns—and more critically due to limited experience and confidence among obstetricians in managing this rare condition (11). Current research primarily focuses on the incidence, risk factors, and adverse maternal and neonatal outcomes associated with face presentation (2, 11–13). However, studies on the rapid diagnosis of sudden face presentations, intrapartum management, and multidisciplinary coordination remain scarce, despite their substantial clinical relevance. The multidisciplinary team (MDT) is an effective medical model that integrates the advantages of multiple clinical specialists for the comprehensive diagnosis and treatment of diseases (14).

**Figure 1:**
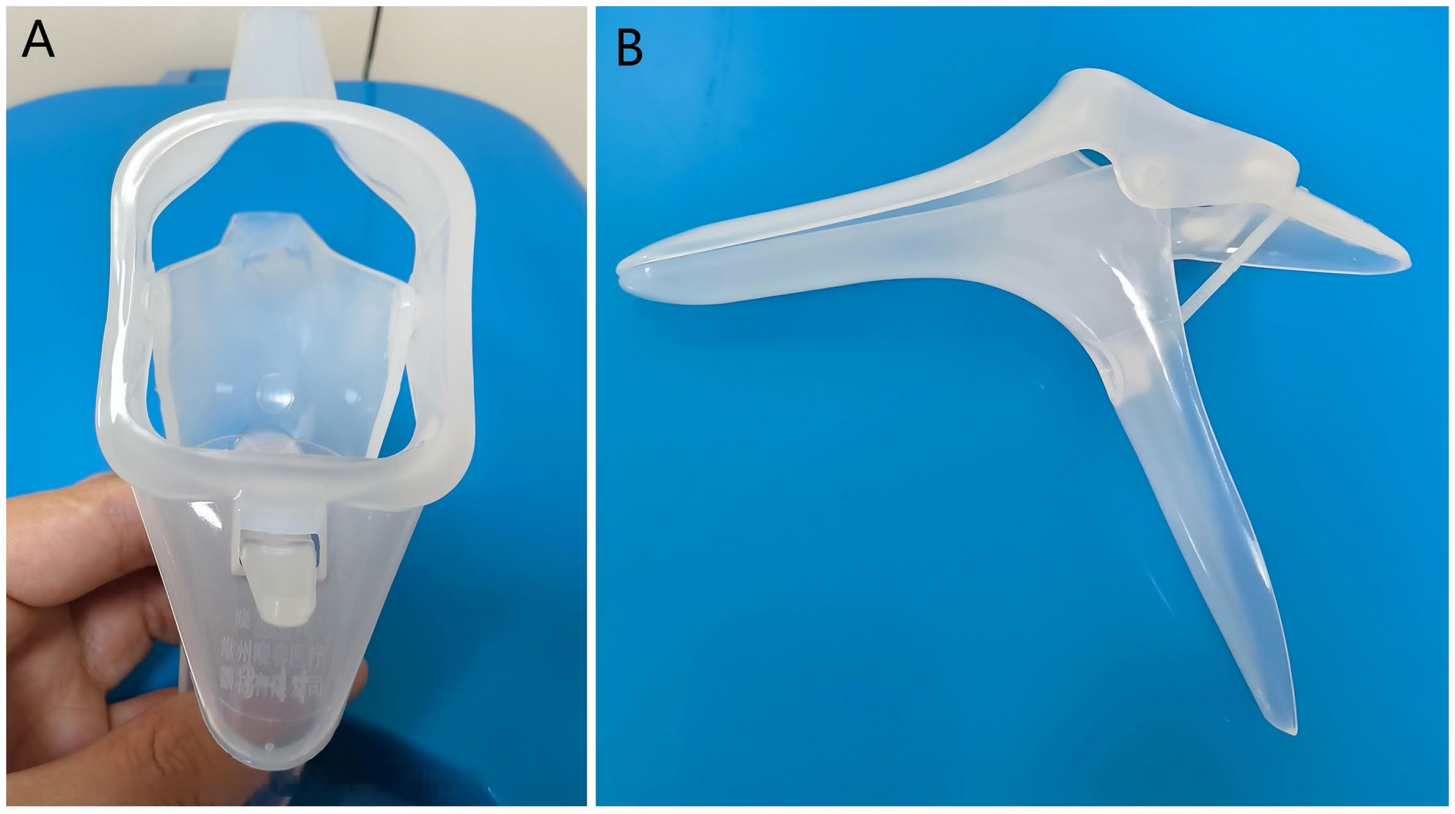
Vaginal dilator. **(A)** Vaginal dilator (front view). **(B)** Vaginal dilator (side view).

The vaginal dilator is a widely used medical device in obstetrics and gynecology, with well-documented applications in the management of vaginismus, post-pelvic radiotherapy care, and postoperative rehabilitation (15–17). Based on their material composition, vaginal dilators are classified into three categories: silicone, plastic, and metal. Silicone dilators, valued for their pliable texture and high biocompatibility, are primarily employed in functional dilation therapy for vaginismus and in the prevention and treatment of vaginal stenosis following pelvic radiotherapy. Plastic dilators, often designed as disposable specula, are intended mainly for diagnostic examination rather than for therapeutic vaginal dilation (Figure 1). At present, metal dilators are rarely used because of their substantial weight, high thermal conductivity, and the need for repeated sterilization. Although characterized by simplicity, cost-effectiveness, and non-invasiveness, the vaginal dilator has seldom been reported in the context of diagnosing face presentation.

This report presents a case of a term pregnant woman who developed a sudden face presentation at the end of the first stage of labor and subsequently underwent successful vaginal delivery, with the aim of informing clinical practice and encouraging further research on managing this uncommon fetal presentation.

## 2 Case presentation

### 2.1 General information

We present the case of a 37-year-old woman at 39 + 5 weeks of gestation, who was admitted on 10 February 2025 with irregular contractions lasting over 2 h for evaluation and delivery. Upon admission, her vital signs were stable: temperature 36.8 °C, pulse 81 bpm, respiratory rate 19 breaths per minute, blood pressure 103/63 mmHg, oxygen saturation 98%, and blood glucose 5.6 mmol/L. Physical examination revealed a uterine height of 36 cm, abdominal circumference of 102 cm, intercristal diameter of 24 cm, intertuberous diameter of 9 cm, external conjugate diameter of 19 cm, and ischial tuberosity diameter of 9 cm. On vaginal examination, the cervix was centrally positioned and soft, with 50% effacement and no dilation. The fetal station was −3, yielding a Bishop score of 4. Ultrasonography revealed a biparietal diameter of 9.29 cm, a fetal heart rate of 143 bpm, and a right occiput posterior (ROP) fetal position. The placenta was attached to the anterior uterine wall, and the amniotic fluid index was 18.4 cm, consistent with a single living fetus. The estimated fetal weight was approximately 3,300 g.

The patient, of advanced maternal age (>35 years), underwent oral glucose tolerance test (OGTT) screening at 24 + 1 weeks, which demonstrated GDM (1-h glucose: 10.23 mmol/L) (18). Her diabetes was diet-controlled, with adjunctive exercise and self-monitoring. Concurrent laboratory testing revealed that the patient was Rh negative. “Obstetric history: Gravida 2, Para 1. One living child, delivered vaginally, weighing 2,600 g.” No history of abortion or induced labor. The remaining laboratory tests, ultrasound examination, and obstetric physical examination showed no significant abnormalities.

Admission diagnosis: The patient was admitted with gestational diabetes mellitus, an Rh-negative blood type. She is gravida two para one (G2P1) at 39 weeks and 5 days gestation with a singleton live fetus presenting with threatened labor.

### 2.2 Diagnosis and delivery process

Day 1 of admission: Due to the patient’s Rh-negative status, a type and screen with crossmatch was completed, and compatible blood units were reserved. An oxytocin challenge test (OCT) was subsequently performed and yielded a negative result.

Day 2 of admission: With a Bishop score of <6, a 10-mg dinoprostone insert was placed for cervical ripening. After approximately 5.5 h, uterine tachysystole developed (11 contractions in 20 min). The insert was promptly removed, and the patient was closely monitored thereafter.

Day 3 of admission: A low-dose oxytocin infusion was administered over 11 h, resulting in improved cervical effacement from 60 to 70%.

**Figure 2:**
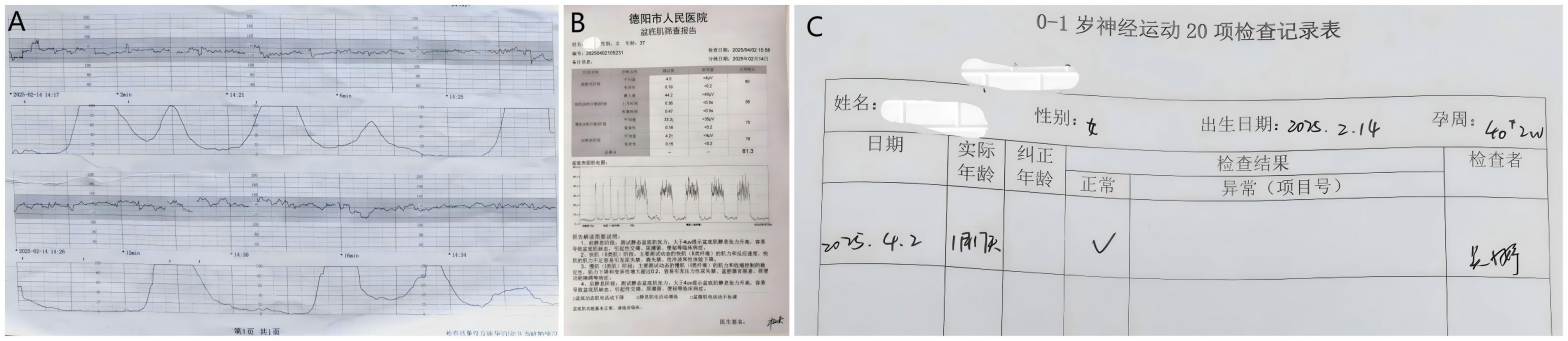
Examination report. **(A)** Fetal monitoring result at 3.5 cm cervical dilatation showing Category I CST. **(B)** Maternal pelvic floor muscle screening report. **(C)** 20-item neuromotor examination report for infants aged 0–1 year.

Day 4 of admission (14 February 2025): The patient remained asymptomatic for labor. With a cervical Bishop score of 5, at 40 + 2 weeks of gestation, and a history of prior vaginal delivery, labor induction was initiated and continued with 2.5 U of oxytocin at 10:04. One hour later, regular uterine contractions began. By 14:30, the cervix had dilated to 3.5 cm, with the presenting part at station −3 (*S* = −3), intact membranes, and an undetermined fetal position. Continuous fetal heart rate monitoring indicated a Category I CST (Figure 2), and the patient continued to await labor progression.

At 17:40, the patient experienced spontaneous rupture of membranes, prompting an immediate vaginal examination. Soft, irregularly shaped fetal parts were palpated at the presenting part, with no cranial sutures or skull bones detectable; the cervix was nearly fully dilated, and the presenting part had descended to station 0 (*S* = 0). Initial assessment suggested a breech presentation. Due to diagnostic uncertainty, the most experienced midwife on duty performed a second vaginal examination. The palpated tissue was found to be uneven, with inconsistent texture and a lip-like structure, raising suspicion of face presentation. However, given the rarity of this presentation and limited diagnostic experience, a definitive diagnosis could not be confirmed by palpation alone. Although ultrasonography provides objective verification, the procedure requires time, potentially delaying critical intervention.

At 17:43, in order to rapidly ascertain the nature of the presenting part, a single-use sterile vaginal dilator (length: 100 mm, speculum width: 38 mm) was placed smoothly following vulvar disinfection. Retraction of the vaginal walls allowed for full exposure of the presenting part, revealing the fetal face with the chin positioned at the 2 o’clock position within the pelvis. Using this method, a diagnosis of left mentum anterior (LMA) face presentation was confirmed within 1 min.

**Figure 3:**
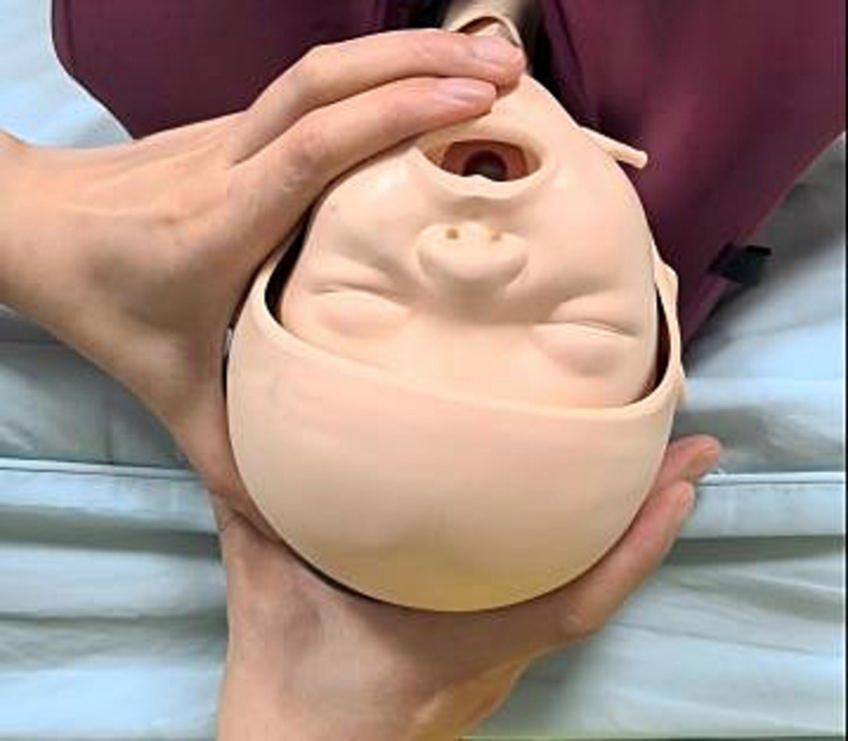
Bimanual escort technique. The left four fingers were positioned on the fetal chin, with the thumb gently pressing the occipital protuberance to ensure that cervical extension did not exceed 160°. The right four fingers supported the perineal body, the palm base supported the levator ani muscle, and the thumb assisted in fetal head flexion.

At 17:45, a comprehensive assessment of pelvic adequacy, uterine contractility, fetal heart rate, and estimated fetal size was undertaken. Simultaneously, the operating room was prepared for a potential emergency cesarean section, and the anesthesia and neonatology teams were placed on standby. During delivery, the midwife employed the “bimanual guidance method” to protect the fetal spine. The bimanual guidance method is an obstetric technique involving coordinated use of both hands to control the fetal head position: the left hand limits cervical extension by pressure on the chin and occiput, while the right hand supports the perineum and assists flexion during delivery (Figure 3). A lateral episiotomy was performed at a 45° angle to the left of the midline of the posterior perineal symphysis as the presenting part descended and perineal tension increased.

At 18:13, the patient delivered vaginally with an estimated blood loss of 240 mL. The neonate had Apgar scores of 10-10-10 and weighed 3,360 g. The patient was transferred to the postpartum ward 2 h later.

On 16 February 2025, at 10:00, both mother and infant had recovered well and were discharged.

On 2 April 2025 (47 days postpartum), the mother and neonate returned for follow-up. The maternal complete blood count and ultrasound were unremarkable. The perineal incision was well-healed. Pelvic floor electromyography revealed mild weakness in Type I muscle fibers with preserved Type II function. Neonatal neuromotor screening was normal (Figure 2). Rh typing for the neonate was deferred to avoid the invasiveness of venipuncture.

## 3 Discussion

Factors that impede fetal head flexion, such as multiparity and fetal malposition, are established risks for face presentation (9, 19, 20). In this case, the patient’s multiparity and the initial right occiput posterior (ROP) position likely predisposed the fetus to incomplete flexion. We propose that the subsequent spontaneous rupture of membranes and rapid fetal descent from station −3 to 0 precipitated acute hyperextension of the fetal cervical spine, leading to the face presentation.

**Figure 4:**
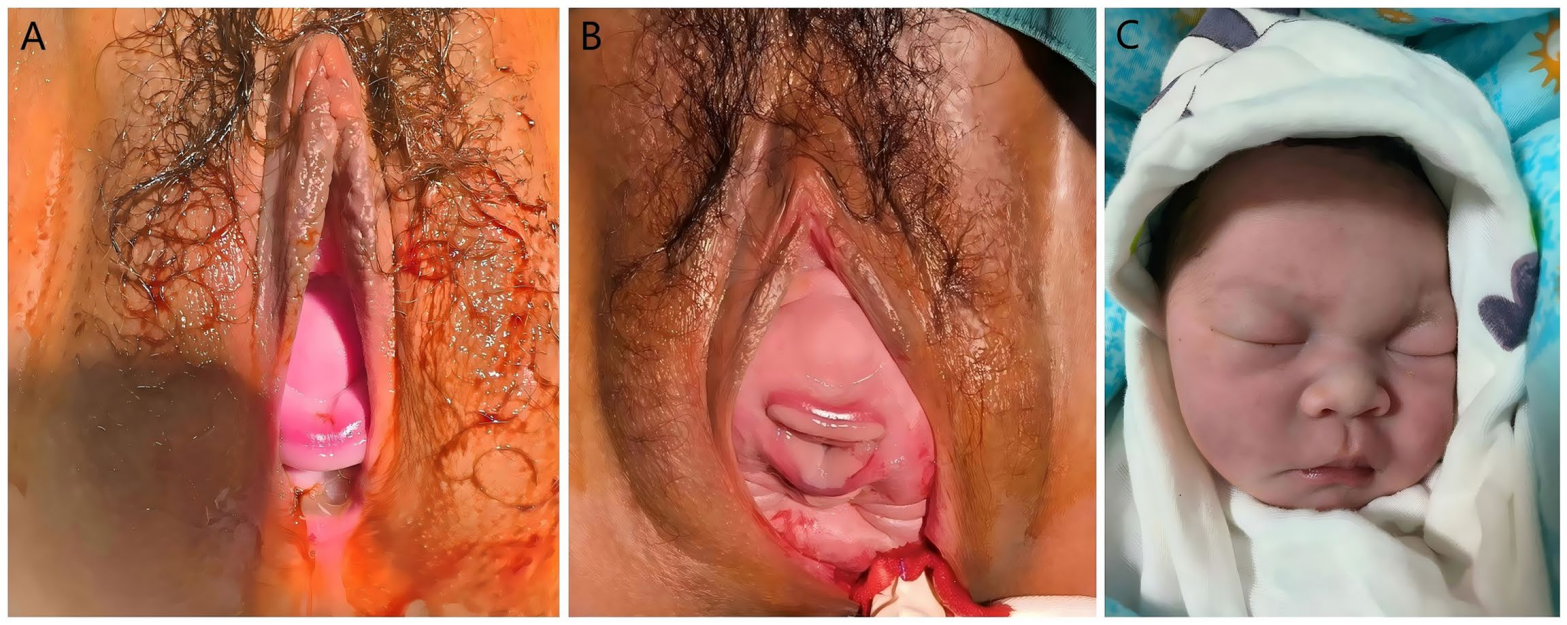
Facial presentation and neonatal facial conditions. **(A)** Fetal chin and lips visible from the vagina. **(B)** Fetal chin, lips, nose, eyes, and forehead visible from the vagina. **(C)** The newborn exhibited no facial edema or ecchymosis, with intact skin integrity at birth.

The mechanism of face presentation differs substantially from that of vertex delivery. In a face presentation, the fetal head extends and descends using the lever formed between the foramen magnum and occiput. Upon encountering pelvic resistance, the fetal head hyperextends—bringing the occiput posteriorly and the chin anteriorly—thus allowing descent with the face as the presenting part (Figure 4). The delivery mechanism in face presentation involves descent with the head in extension, which engages a larger and less malleable diameter than in vertex presentation. This can prolong labor and increase the risk of both maternal and fetal complications. Potential adverse outcomes include severe perineal lacerations, fetal distress, and, in cases of excessive hyperextension, fetal spinal cord injury (10, 21–23). These risks underscore the need for timely and accurate diagnosis (see Table 1).

**Table 1:** Patient care timeline (admission → follow-up).

Date	Time	Event
10 February 2025	9:00	Admission
14 February 2025	10:04	Oxytocin with a low dose
	11:04	Regular contractions
	14:30	Cervix dilated to 3.5 cm with intact membrane
	17:40	Spontaneous rupture of amniotic fluid, soft tissue extrusion palpated, breech presentation suspected
	17:43	Vaginal dilators used
	17:44	Confirmed face presentation
	18:13	Uneventful delivery
16 February 2025	10:00	Mother and infant recovered well and were discharged
02 April 2025		Mother and infant returned for re-examination

Digital vaginal examination is the standard for diagnosing face presentation, but its accuracy is limited by operator experience and can be confounded by caput succedaneum, as observed in this case, where two examinations were inconclusive. While intrapartum ultrasound is a reliable alternative, it may not be immediately available, potentially delaying management (6). We employed a sterile vaginal dilator for direct visualization of the presenting part. This allowed for definitive identification of facial landmarks and confirmation of a left mentum anterior (LMA) position in under 1 min, demonstrating a practical method for rapid diagnosis when palpation is uncertain.

The use of a vaginal dilator for diagnosis is a simple technique that requires minimal equipment and may be particularly useful in settings where immediate ultrasound is unavailable. Its utility is likely greatest in the late first or second stage of labor when the cervix is sufficiently dilated and the presenting part is engaged. Certain limitations exist during early labor—if cervical dilation is inadequate or the fetal station is high, speculum examination may become technically difficult and less reliable. Following diagnosis, our institutional protocol for obstetric emergencies was activated, ensuring that anesthesia, neonatology, and operating room staff were on standby for a potential emergency cesarean delivery. This multidisciplinary readiness is a key component of safe management for high-risk intrapartum events.

During the second stage, the “bimanual guidance method” was used to control delivery of the head. This technique, involving one hand on the fetal chin and occiput to moderate extension and the other supporting the perineum, is intended to prevent rapid, uncontrolled delivery and potential spinal hyperextension. An episiotomy was performed to reduce perineal resistance as the head crowned. The neonate exhibited normal limb movement without significant facial edema or ecchymosis (Figure 4), and no complications were noted at the 47-day follow-up.

Effective communication and a coordinated team response were noted by the patient as important factors in alleviating her anxiety during this unexpected event, reinforcing the value of a patient-centered approach during obstetric emergencies. As an expression of gratitude, she presented the team with a banner of appreciation.

This case report describes the successful vaginal delivery of a term fetus in a face presentation that was diagnosed intrapartum using direct visualization with a vaginal dilator. This technique proved to be a rapid and effective adjunct when digital examination was inconclusive. We also describe a bimanual guidance method for fetal spinal protection during delivery.

While the outcomes were favorable, this report is limited to a single case. The efficacy and safety of these techniques, particularly the use of a vaginal dilator for diagnosis, require evaluation in larger studies to determine their role in the management of face presentation.

## Data Availability

The original contributions presented in the study are included in the article/Supplementary material, further inquiries can be directed to the corresponding author.
