# Supplementary material for: Diagnosis and management of face presentation: a case report featuring an innovative diagnostic approach and fetal spinal protection technique
Source: Front Med (Lausanne). 2025 Sep 26;12:1664796. doi: 10.3389/fmed.2025.1664796 (PMC12511126; doi:10.3389/fmed.2025.1664796)
Supplement: Supplementary file 1 [file Table_1.DOCX]

Supplementary Material

# Table

****Patient Care Timeline (Admission → Follow-up)****

| ****Date**** | ****Time**** | ****Event**** |
| --- | --- | --- |
| ****10 February 2025**** | ****9:00**** | ****Admission**** |
| ****14 February 2025**** | ****10:04**** | ****Oxytocin with low dose**** |
|  | ****11：04**** | ****Regular contractions**** |
|  | ****14：30**** | ****Cervix dilated to 3.5 cm with intact membranes**** |
|  | ****17：40**** | ****Spontaneous rupture of amniotic fluid, soft tissue extrusion palpated, breech presentation suspected**** |
|  | ****17：43**** | ****Vaginal dilators used**** |
|  | ****17：44**** | ****Confirmed face presentation**** |
|  | ****18：13**** | ****Uneventful delivery**** |
| ****16 February 2025**** | ****10：00**** | ****Mother and infant recovered well and were discharged**** |
| ****02 April 2025**** |  | ****Mother and infant return for reexamination**** |

**
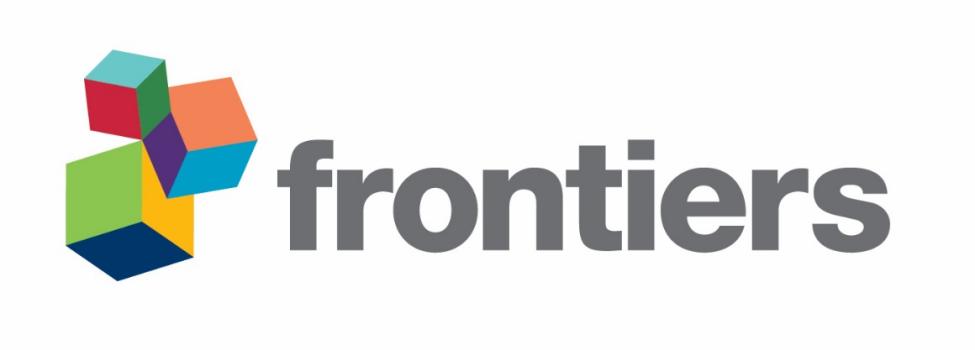
**
